# An interferon signature is associated with HAM/TSP but not viral containment in HTLV-1 infection

**DOI:** 10.1186/1742-4690-8-S1-A108

**Published:** 2011-06-06

**Authors:** Sonja Tattermusch, Jason Skinner, Damien Chaussabel, Jacques Banchereau, Matthew P Berry, Anne O'Garra, Graham P Taylor, Charles R M Bangham

**Affiliations:** 1 Departments of Immunology and GU Medicine and Communicable Diseases, Imperial College London, London, W2 1PG, UK; 2 Baylor Institute for Immunology Research, INSERM U-899, Dallas, TX 75204, USA; 3 Division of Immunoregulation, MRC National Institute for Medical Research, London, NW7 1AA, UK

## 

Most people infected with Human T-cell Lymphotropic Virus Type 1 (HTLV-1) remain clinically asymptomatic; however, a minority develops the debilitating myelopathy HAM/TSP. Current treatment of HAM/TSP is limited by our partial understanding of the protective immune response to HTLV-1 and the pathogenesis of HAM/TSP.

We wished to test the hypothesis that a gene expression signature in peripheral blood distinguishes between patients with HAM/TSP and ACs. We investigated genome-wide transcription patterns in whole blood from HTLV-1 asymptomatic carriers (AC; n=37), patients with HAM/TSP (n=20) and uninfected control subjects (n=17). We identified a 542-gene signature that was deregulated in all HTLV-1+ individuals and predominantly comprised transcripts involved in p53-mediated DNA damage responses (p=0.00489). An 80-gene signature distinguished patients with HAM/TSP from those with the clinically similar disease multiple sclerosis. Paradoxically, at a given proviral load patients with HAM/TSP, but not ACs, over-expressed antiviral interferon-stimulated genes (ISGs; p=0.00859).

Expression of these ISGs (assessed by quantitative PCR and flow cytometry) was not limited to HTLV-1-infected CD4+ T cells, suggesting that all peripheral blood immune cells were exposed to interferons (IFN) in vivo. Neither elevated IFN plasma levels nor an abnormal capacity for IFN production was detected in patients with HAM/TSP. However, peripheral immune cells in patients with HAM/TSP were more sensitive to IFN-alpha and IFN-gamma stimulation.

These findings suggest that chronic over-expression of a specific subset of ISGs is ineffective in containing HTLV-1 and may instead contribute to the pathogenesis of HTLV-1-associated myelopathy.

